# Anxiety and its influence on maternal breastfeeding self-efficacy*

**DOI:** 10.1590/1518-8345.5104.3485

**Published:** 2021-11-08

**Authors:** Luciana Camargo de Oliveira Melo, Marina Cortez Pereira Bonelli, Rosa Vanessa Alves Lima, Flávia Azevedo Gomes-Sponholz, Juliana Cristina dos Santos Monteiro

**Affiliations:** 1 Universidade de São Paulo, Escola de Enfermagem de Ribeirão Preto, PAHO/WHO Collaborating Centre for Nursing Research Development, Ribeirão Preto, SP, Brazil.

**Keywords:** Breast Feeding, Anxiety, Self-Efficacy, Nursing Research, Maternal and Child Health, Mental Health, Lactancia Materna, Ansiedad, Autoeficacia, Investigación em Enfermería, Salud Materno-Infantil, Salud Mental, Aleitamento Materno, Ansiedade, Autoeficácia, Pesquisa em Enfermagem, Saúde Materno-Infantil, Saúde Mental

## Abstract

**Objective:**

to identify the levels of anxiety and breastfeeding self-efficacy in puerperal women at the intervals of 60, 120, and 180 days postpartum; and to verify the influence of anxiety on breastfeeding self-efficacy among these puerperal women.

**Method:**

an analytical, longitudinal and prospective study, conducted with 186 puerperal women, and which used a sociodemographic and obstetric characterization questionnaire, the State-Trait Anxiety Inventory, and the Breastfeeding Self-Efficacy Scale - Short Form. The analyses were performed by means of descriptive statistics, and Fisher’s Exact Test was used.

**Results:**

most of the puerperal women presented low levels of trait anxiety (55.4%) and of state anxiety (66.2% at 60 days, 72.8% at 120 days, and 75.5% at 180 days), and a high level of self-efficacy (77.3% at 60 days, 75.3% at 120 days, and 72.1% at 180 days of puerperium). Low levels of trait anxiety and state anxiety were associated with high self-efficacy at 60 days (p=0.0142 and p=0.0159, respectively).

**Conclusion:**

it is necessary to pay greater attention to the mental health of puerperal women, considering that those who presented low levels of anxiety had higher levels of self-efficacy, a situation that can result in longer exclusive breastfeeding.

## Abstract (es)

**Objetivo:**

identificar los niveles de ansiedad y autoeficacia para la lactancia materna en puérperas a intervalos de 60, 120 y 180 días posparto; verificar la influencia de la ansiedad sobre la autoeficacia para amamantamiento en estas madres.

**Método:**

estudio analítico, longitudinal y prospectivo, con 186 puérperas, utilizando un cuestionario sociodemográfico y obstétrico, el Inventario de Ansiedad Rasgo-Estado y la *Breastfeeding Self-Eficcacy Scale - Short Form*. Los análisis se realizaron mediante estadística descriptiva y se utilizó la prueba exacta de Fisher.

**Resultados:**

la mayoría de las puérperas presentaron bajo nivel de ansiedad rasgo (55,4%), bajo nivel de ansiedad estado (66,2% a los 60 días, 72,8% a los 120 días y 75,5% a los 180 días) y alto nivel de autoeficacia (77,3% a los 60 días, 75,3% a los 120 días y 72,1% a los 180 días del puerperio). Los niveles bajos de ansiedad rasgo y ansiedad estado se asociaron con una alta autoeficacia a los 60 días (p=0,0142 y p=0,0159 respectivamente).

**Conclusión:**

es necesario prestarle mayor atención a la salud mental de las puérperas, dado que las mujeres que presentaban bajos niveles de ansiedad tenían mayores niveles de autoeficacia, lo que puede implicar un mayor tiempo de lactancia materna exclusiva.

## Abstract (pt)

**Objetivo:**

identificar os níveis de ansiedade e da autoeficácia para amamentação entre puérperas nos intervalos de 60, 120 e 180 dias pós-parto; verificar a influência da ansiedade na autoeficácia para amamentação entre essas puérperas.

**Método:**

estudo analítico, longitudinal e prospectivo, com 186 puérperas, utilizando-se um questionário de caracterização sociodemográfica e obstétrica, o Inventário de Ansiedade Traço-Estado e a *Breastfeeding Self-Eficcacy Scale - Short Form*. As análises foram feitas pela estatística descritiva, e utilizado o Teste Exato de Fisher.

**Resultados:**

a maioria das puérperas apresentou baixo nível de ansiedade-traço (55,4%), baixo nível de ansiedade-estado (66,2% aos 60 dias, 72,8% aos 120 dias e 75,5% aos 180 dias) e alto nível de autoeficácia (77,3% aos 60 dias, 75,3% aos 120 dias e 72,1% aos 180 dias de puerpério). Baixos níveis de ansiedade-traço e de ansiedade-estado associaram-se com a autoeficácia elevada aos 60 dias (p=0,0142 e p=0,0159 respectivamente).

**Conclusão:**

é necessária uma maior atenção à saúde mental das puérperas, considerando que mulheres que apresentaram baixos níveis de ansiedade tiveram maiores níveis de autoeficácia, situação que pode implicar em maior tempo de aleitamento materno exclusivo.

## Introduction

Adequate nutrition during childhood is considered essential and of great repercussion for reaching better health, nutrition, growth, development, and surviving conditions of the infant, a stage in which breastfeeding stands out as the ideal nutrition source^(1-2)^. Breastfeeding is the most efficient action, for it has repercussions in the comprehensive health of both mother and child, in addition to nurturing, reinforcing the bond, and promoting affection and protection, impacting on the health indicators of the entire society^(2-3)^.

The National Study on Infant Feeding and Nurturing (*Estudo Nacional de Alimentação e Nutrição Infantil*, ENANI), which is the most recent research study conducted about breastfeeding indicators in Brazil, observed in its preliminary results an increase in all the prevalence values of this practice in children under five years of age^(4)^. However, the results showed that we have not yet attained what is proposed by the international and national health institutions^(3)^.

Despite the scientifically proven evidence of the countless benefits of breastfeeding, a number of studies reinforce that its early interruption still represents a contradiction in Brazil and worldwide^(5)^. Considering that the puerperium is a relevant period during the woman’s life, in which several and significant physiological, psychological, and social transformations occur, it becomes fundamental to monitor the woman’s health in this period^(6)^. Thus, the literature emphasizes the involvement of psychosocial factors, such as maternal breastfeeding self-efficacy and anxiety^(7)^, as strong determinants for the breastfeeding practice. Maternal breastfeeding self-efficacy is a concept defined as the woman’s conviction or trust about her potential or skill to successfully conduct the breastfeeding practice^(8)^. Women who trust in their ability to breastfeed do so for a longer period when compared to those who lack such self-confidence, turning self-efficacy into an important protective factor for breastfeeding^(9)^.

Anxiety is defined as a natural and adaptive reaction that can trigger physiological, behavioral and affective reactions, as well as exert an influence on the behaviors. However, it starts to be considered as pathological when those referred feelings are inharmonious to the event that triggers it, or when there is not any specific cause or reason for its emergence^(10)^.

A number of studies indicate that high anxiety rates in the puerperium are associated with greater chances of not initiating and continuing breastfeeding and, also, of complementation by using milk formulas^(11-12)^. A prospective study revealed a statistical and clinically significant relationship between the anxiety levels and the reduction in breastfeeding duration, highlighting that even the low levels of anxiety should not be neglected because they can be associated with early weaning^(13)^.

Despite the diverse evidence indicating that high self-efficacy levels have the power to positively influence the maintenance of breastfeeding^(14)^, and that the presence of anxiety can negatively influence this practice^(15)^, studies that evaluate the relationship of those variables were not found in the scientific literature. That fact shows that important failures persist, which justifies the conduction of this study. Our hypothesis is that the presence of some anxiety disorder indicator influences the level of breastfeeding self-efficacy.

This study has the following objectives: to identify the levels of anxiety and breastfeeding self-efficacy in puerperal women at intervals of 60, 120, and 180 days postpartum; and to verify the influence of anxiety on breastfeeding self-efficacy among these puerperal women.

## Method

### Study type

This is an analytical, longitudinal and prospective study.

### Data collection locus

Data collection took place in a Basic Health Unit (BHU) of a large city in the inland of São Paulo, Brazil.

### Period

The data collection period was between October 2018 and July 2019.

### Population and sample

The study population consists of all women at 60 days postpartum, and who were present for the monitoring of their child in the childcare consultation or for vaccination in the BHU. Those same women were again interviewed when they were at 120 days and 180 days postpartum, using the same instruments at the three moments. Sample calculation was done based on information from the 2017 Annual Report of the aforementioned Health Unit and on previous research studies involving maternal breastfeeding self-efficacy and mental health (indicators of trait anxiety and state anxiety symptoms). Selection of the study participants was by simple random sampling, a 5% significance level being considered for the statistical calculation, as well as a statistical power of 95%, thus composing a sample of 180 women at a first moment (60 days of puerperium), already considering a predicted loss of 10%.

### Selection criteria

The inclusion criteria established were established as follows: women over 18 years old, who were breastfeeding at 60 days, 120 days, and 180 days postpartum. Exclusion criteria were also established, namely: women who had any type of hearing and/or visual impairment which made it impossible for them to answer the instruments, as well as women whose children were receiving any type of special care and/or presented any anomaly or malformation.

### Participants

A total of 186 puerperal women took part in the first moment of data collection, 162 in the second moment, and 147 women in the third moment.

### Study variables

Dependent variable: level of maternal self-confidence in breastfeeding during the puerperium. Independent variables: the presence of anxiety as a trait: low, medium, high; the presence of anxiety as a state: low, medium, high. Variables related to the characterization of the participants: age, skin color, schooling, religion, marital status, occupation, income, help with caring for the infant, and type of breastfeeding during data collection.

### Instruments used to collect the information

Three instruments were used for data collection: the first one contemplated identification and qualification data of the women participating in the study, elaborated and designed by the researcher, based on the national and international scientific literature. The second instrument, the State-Trait Anxiety Inventory (STAI), is a public domain instrument validated, translated, and adapted into more than 30 languages in several countries, including Brazil^(16)^. The STAI was chosen because it is an instrument that has already used in several clinical studies, including during the puerperium, proving to be very sensible and practical in identifying anxiety, even not being a specific instrument for this period^(16)^.

The STAI is composed of two scales that measure two differentiated concepts of anxiety: state anxiety (STAI-S) and trait anxiety (STAI-T). State anxiety refers to a one-time period or circumstance, it is a limited or temporary situation, depending on the context, being considered variable over time. Trait anxiety refers to the particular characteristics of the individual, it is stable and constant, differentiating the individual’s response in the face of diverse situations, and is considered a permanent characteristic of the individual. STAI-S and STAI-T contain 20 items each with four answer choices whose values vary from one to four points. To dimension and interpret the answers, scores corresponding to the values between 1 “almost never” and 4 “almost always” are assigned. The total sum of each question’s points results in a minimum score of 20 and a maximum of 80 in each scale, with results from 20 to 40 points meaning low anxiety level; from 41 to 60 points, medium anxiety level; and from 60 to 80 points, high anxiety level. For calculation, some questions go through item recoding, that is, in case the answer is 4, a value of 1 is assigned to it in the coding; for a 3 answer, a value of 2 is assigned; if the answer is 2, 3 is assigned; and, if it is 1, 4 is assigned.

The third instrument is the Brazilian version of the Breastfeeding Self-Efficacy Scale - Short Form (BSES-SF), for assessing breastfeeding self-efficacy, validated in a study developed in Brazil with puerperal women in a public maternity hospital^(17)^, used in this study under authorization by the researcher. Based on the psychometric tests performed for the validation of this scale, it was proven that it can be used in various cultures and age ranges^(17)^.

The self-efficacy values for breastfeeding obtained by means of the scale are set according to the referred values, and the total scale with 14 items, with a variation from 1 to 5 points for each item, presents a minimum score of 14 and a maximum score of 70 points. Thus, the BSES-SF considers the following: low self-efficacy, from 14 to 32 points; medium self-efficacy, from 33 to 51 points; and high self-efficacy, from 52 to 70 points^(17)^.

### Data collection

The data were obtained by the researcher herself at three different moments in a reserved room in the aforementioned health unit. The data collection instruments were applied, and completion lasted a mean of 15 minutes. At the first moment (60 days postpartum): when the women went to the health unit accompanying their children to attend the childcare consultation and/or 2^nd^-month child’s vaccination, STAI-trait, STAI-state, and BSES-SF were applied. At the second moment (120 days postpartum): when the women went to the health unit accompanying their children to attend the childcare consultation and/or 4^th^-month child’s vaccination, STAI-state and BSES-SF were applied. At the third moment (180 days postpartum): when the women went to the health unit accompanying their children to attend the childcare consultation and/or child vaccination at the 6^th^ month of life, STAI-state and BSES-SF were applied.

### Data treatment and analysis

The participants’ characterization was based on descriptive statistics by using the mean and median calculations, standard deviations, minimum and maximum, indicating data variability. To verify the association between the variables, Fisher’s Exact Test was used and a 5% significance level was adopted (alpha = 0.05). The statistical program used was the R program (R Core Team, 2018), version 3.6.1.

It was necessary to group the values obtained for the self-efficacy variables (medium and low) when they were associated with the anxiety (trait and state) variable for visualizing this association. The scores obtained by the participants for low self-efficacy were zero at all research moments, that is, no participant presented low self-efficacy.

### Ethical aspects

The research was approved by the Research Ethics Committee linked to the National Research Ethics Commission (*Comissão Nacional de* Ética *em Pesquisa*, CONEP) with CAAE protocol No. 92340718.0.0000.5393.

## Results

A total of 186 puerperal women participated in this study at the first moment, 162 at the second moment, and 147 at the third moment; the mean age was 26 years old, with a minimum of 18 years old, a maximum of 47 years old, and a standard deviation of 6.25. Regarding skin color, brown skin color was referred to by 60.8% of the total participants. In the schooling item, 43.0% of the puerperal women referred having completed High School, and most of them stated professing some religion (76.3%). Regarding occupation, 49.5% of the participants stated that they perform some paid work outside their homes. Regarding monthly family income, the mean among the women who declared income was R$ 2,300.45. Regarding marital status, most reported having a partner; of those, 55.9% were in a stable union and 32.3% were married. In relation to receiving help from someone in caring for the baby, most of the women reported such assistance, with 53.8% receiving from her husband. Of the total participants, 69.9% were exclusively breastfeeding (EMB) at 60 days of puerperium, 51.2% at 120 days, and 25.9% at 180 days.

Regarding self-efficacy, it was observed that most of the women obtained a high level for this variable at the three moments studied, and that rate remained with very similar values at all research moments, as shown in Table 1.

**Table 1:** Distribution of the participants regarding the breastfeeding self-efficacy classification, according to days of puerperium. Ribeirão Preto, SP, Brazil, 2018-2019

Breastfeeding self-efficacy							
Time	High		Medium		Low		Total
	Frequency	%	Frequency	%	Frequency	%	
60 days	144	77.3	42	22.6	1	0.5	186
120 days	122	75.3	40	24.7	0	0	162
180 days	106	72.1	41	27.9	0	0	147

Regarding the anxiety disorder, the value obtained by the participants in relation to the answers to the STAI showed that most of the women presented a low level of trait anxiety, as shown in Table 2.

**Table 2:** Distribution of the study participants according to the trait anxiety classification at 60 days of puerperium (n=186). Ribeirão Preto, SP, Brazil, 2018-2019

Trait anxiety		
Classification	Frequency	%
High	11	5.9
Medium	72	38.7
Low	103	55.4

Table 3 shows the results of the values obtained by the participants with regard to the level of state anxiety. It is observed that most of the women had a low anxiety level at the three moments studied.

**Table 3:** Distribution of the study participants regarding the state anxiety classification according to days of puerperium. Ribeirão Preto, SP, Brazil, 2018-2019

State anxiety							
Time	High		Medium		Low		Total
	Frequency	%	Frequency	%	Frequency	%	
60 days	3	1.6	60	32.2	123	66.2	186
120 days	1	0.6	43	26.6	118	72.8	162
180 days	1	0.6	35	23.9	111	75.5	147

Table 4 presents the results of the association between breastfeeding self-efficacy and trait anxiety, showing that the puerperal women who obtained a low anxiety level presented a higher level of breastfeeding self-efficacy, and this result was considered as statistically significant (p=0.0142).

**Table 4:** Analysis of breastfeeding self-efficacy associated with the trait anxiety classification at 60 days of puerperium (n=186). Ribeirão Preto, SP, Brazil, 2018-2019

Breastfeeding self-efficacy						
Time	STAI-trait	High		Medium/Low		p-value*
		Frequency	%	Frequency	%	
60 days	High	6	3.20	5	2.60	0.0142
	Medium	51	27.5	21	11.3	
	Low	87	46.8	16	8.60	

* Fisher’s exact test

In the analysis of the association between self-efficacy and state anxiety, the results demonstrate that after 60 days of puerperium, the participants who presented low levels of state anxiety obtained a higher level of breastfeeding self-efficacy, and this analysis was statistically significant (p= 0.0159), as described in Table 5.

**Table 5:** Analysis of breastfeeding self-efficacy associated with the state anxiety classification according to days of puerperium. Ribeirão Preto, SP, Brazil, 2018-2019

Breastfeeding self-efficacy						
Time	STAI-state	High		Medium/Low		p-value*
		Frequency	%	Frequency	%	
60 days	High/Medium	42	22.6	21	11.3	0.0159
	Low	102	54.8	21	11.3	
120 days	High/Medium	28	17.3	14	8.7	0.1482
	Low	94	58	26	16	
180 days	High/Medium	24	16.3	8	5.3	0.8248
	Low	83	56	33	22.4	

* Fisher’s Exact Test

## Discussion

The breastfeeding self-efficacy levels obtained by most of the participants in this study were considered high at the three moments, with very similar scores, varying from 77.3% at the first moment to 72.1% at the third research moments. Only one woman presented low self-efficacy at the first moment, and no puerperal woman presented that rate at any other moment.

A study on breastfeeding self-efficacy carried out with puerperal women showed results similar to those of this study in 53.4% of the participants^(18)^.

However, despite high levels of self-efficacy registered after delivery are considered to contribute to better acceptance and adherence to this practice, there is no scientific evidence that high self-efficacy ensures its continuity for a 6-month period or more, given that self-efficacy depends on its four pillars that are variable throughout the days: due to personal experience; due to living together with women who are breastfeeding or had already breastfed; due to the knowledge shared with their support relationship network; and due to their emotional and physical state^(19)^. In this perspective, early identification in puerperal women of factors involved in the breastfeeding process can assist in specific interventions aiming to keep EMB for longer periods of time^(20)^.

It is understood from the results found that the women who took part in this study maintained their breastfeeding self-efficacy high, regardless of the period approached. According to the literature in this regard, the result can be related to previous factors, such as guidelines offered during prenatal care and experiences lived, as well as pro-breastfeeding activities carried out in the health units, being essential for the maintenance of high breastfeeding self-efficacy values in the women and, consequently, better breastfeeding indicators^(21-22)^.

Anxiety during the puerperium can be considered as an alarming factor, for it negatively interferes in the affective bond between the mother and her child, reducing the women’s ability to cope with the transformations inherent to the period, with the possibility of worsening throughout the child’s growth^(23)^. The literature points out that mothers who present anxiety symptoms report greater complexity to understand their own emotions, which can directly and negatively interfere with the provision of care to the child^(24)^.

In this study, the results showed that most of the women presented a low level of trait anxiety (55.4%) and also of state anxiety at the three moments investigated (66.2%, 72.8%, and 75.5%). It is suggested that the results are related to the characteristics presented by the study participants, and that they are protective factors for the mental health of these women. Other research studies have shown that women without a partner, with lower schooling, not performing any paid work and with a family income of less than R$ 1,000.00, present higher anxiety levels when compared to the group of women that did not report the same characteristics, corroborating our results and those of other studies in the national and international scope^(25-26)^.

The results of the association between breastfeeding self-efficacy and trait anxiety showed that the puerperal women who obtained a low level of trait anxiety presented a higher level of breastfeeding self-efficacy, this result being statistically significant (p=0.0142). In the analysis of the association between self-efficacy and state anxiety, the results showed that, at 60 days of puerperium, the participants that presented low state anxiety scores had a higher level of breastfeeding self-efficacy (p=0.0159). No studies were found that assessed the association between self-efficacy and anxiety. By detecting the conditions that may influence the values of breastfeeding self-efficacy, such as anxiety herein described, health professionals can strategically work with the women and intervene early to promote breastfeeding^(27)^.

Although the study municipality does not have a specific program aimed at women who present mental disorders, or does not even screen breastfeeding self-efficacy, it can be inferred that the care provided by the professionals to the participants in this study is articulated in such a way that it indicates that the professional practice is based beyond the competences regarding the physiology and technique to handle breastfeeding, which can be confirmed by the values found in this study, of the EMB prevalence at 60 days and at 120 days. The actions performed by the health professionals are articulated considering possible mental disorders beyond the biological, social, and emotional fields, reinforcing the appreciation of the women’s skills and experiences during the breastfeeding process.

Thus, it can be understood that the outcome found in this study can be the result of actions referring to the changes in the professional practice and to the reorganization of the work process, referred to by the basic health conditions of the population in line with the principles established by the Breastfeed and Feed Brazil Strategy, an important public policy to encourage breastfeeding that was adopted by this municipality.

One of the limitations of this study was the fact that the presence of anxiety symptoms was not investigated, or any treatment previous to data collection. There was also the unavailability to monitor those participants who were identified with high anxiety levels according to the scale applied and forwarded for better assessment and course of action according to the BHU’s protocols. However, the limitations do not imply any loss for the study, for its objective contemplated the identification of anxiety levels, regardless of those factors.

## Conclusion

This paper confirmed the hypothesis raised and concluded that low anxiety levels were associated with higher levels of breastfeeding self-efficacy at 60 days of puerperium, a situation that can result in longer exclusive breastfeeding. Efforts should be made to reduce the chances of early weaning and to improve the mental health of women in the postpartum period, thus contributing to better indicators for the life condition of the women and her baby in the breastfeeding process, assisting in the consolidation of the benefits of this practice so that they reflect on the entire society.

We highlight the importance of developing new and more in-depth research studies in this area, adding the results obtained in this research, given that anxiety and self-efficacy can bring about implications to the maternal experience, the relationship between the women and her children, as well as to the children’s development. The analysis of the results in this study can assist in the clinical practice scope, the professional behavior in the sense of overcoming the informative conception of health care, in addition to more comprehensively understanding the pregnancy-puerperal cycle specificities.
